# Forensic SNP Genotyping using Nanopore MinION Sequencing

**DOI:** 10.1038/srep41759

**Published:** 2017-02-03

**Authors:** Senne Cornelis, Yannick Gansemans, Lieselot Deleye, Dieter Deforce, Filip Van Nieuwerburgh

**Affiliations:** 1 Laboratory of Pharmaceutical Biotechnology, Ghent University Ghent, Belgium.

## Abstract

One of the latest developments in next generation sequencing is the Oxford Nanopore Technologies’ (ONT) MinION nanopore sequencer. We studied the applicability of this system to perform forensic genotyping of the forensic female DNA standard 9947 A using the 52 SNP-plex assay developed by the SNPforID consortium. All but one of the loci were correctly genotyped. Several SNP loci were identified as problematic for correct and robust genotyping using nanopore sequencing. All these loci contained homopolymers in the sequence flanking the forensic SNP and most of them were already reported as problematic in studies using other sequencing technologies. When these problematic loci are avoided, correct forensic genotyping using nanopore sequencing is technically feasible.

Short tandem repeats (STRs) have been the golden standard in forensic DNA casework for many years. However, single nucleotide polymorphism (SNP) typing holds some advantages over STR analysis in forensics and paternity testing^1^,^2^,^3^. The ability to design an assay based on very small amplicons allows for improved detection in highly degraded samples^4^. Moreover, the SNP mutation rate is significantly lower, which is an advantage in kinship analysis^2^. However, as most SNPs are bi-allelic, this significantly reduces their discrimination power^3^. In order to obtain forensic power comparable to conventional STR based assays, a higher number of SNP loci is required^5^. The SNPforID consortium developed a 52-SNP multiplex which matches the discrimination power of routinely used 10–15 STR multiplexes^1^. This SNP multiplex was initially designed to be analyzed via Single Base Extension (SBE) combined with capillary electrophoresis (CE)^6^. Although proficient, some issues inherent to this technique hampered its global implementation and led to the development of several other detection methods, such as mass spectrometry, microarray hybridization, oligo ligation assays and pyrosequencing^7^. The use of massive parallel sequencing (MPS) can be an attractive alternative to the PCR-SBE-CE workflow. MPS technologies allow high throughput sequencing of a large pool of amplicons in a single experiment enabling variant polymorphism detection with single base pair resolution. Recently, Oxford Nanopore developed a pocketsize MPS device called MinION. This low cost, highly portable sequencer uses nanoscopic pores through which DNA strands are translocated. The ionic current associated with this process is measured and used to identify the nucleotides passing through the pore. The quasi real time strand sequencing allows for an on-the-fly base calling and a ‘run until’ analysis, i.e. running the device until a required amount of data has been produced. In this proof-of-principle study the potential of the MinION sequencer to generate data allowing the deduction of a 52 SNP profile was investigated. Verification was done by comparison to the profile obtained via Illumina sequencing. Based on our results, a first assessment of the performance of the MinION sequencer to analyze a forensic SNP multiplex, as well as identifying criteria for selecting MinION compatible SNP markers was made.

## Results and Discussion

**Figure 1:**
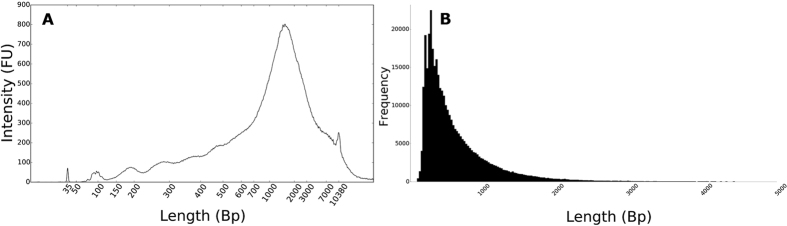
(**A**) Length profile (bp) of concatenated amplicons as measured with an Agilent High-Sensitivity DNA chip; internal marker at 35 bp and 10380 bp. (**B**) Read length (bp) histogram of the high quality two-directional (2D) nanopore reads.

The SNP amplicon ligation protocol produced DNA fragments with a median length of around 1000–2000 bp (Fig. 1A), thereby clearly overcoming the minimum length requirement (100 bp) set by the Metrichor base calling software. Without this concatenation step, only 12 of the 52 loci amplicons would meet the minimum length requirements. The 24 h sequencing run generated a total of 776816 reads, of which 367920 were categorized by Metrichor as high quality two-directional (2D) reads. Two-directional reads are based on the sequencing of the template and the complement strand of a double stranded DNA fragment and are thus more accurate because the reads are generated using the combined nanopore signal from the template strand and the complement strand. The 2D reads had a mean read length of 625 bp and the longest read measured 13380 bp (Fig. 1B).

**Figure 2:**
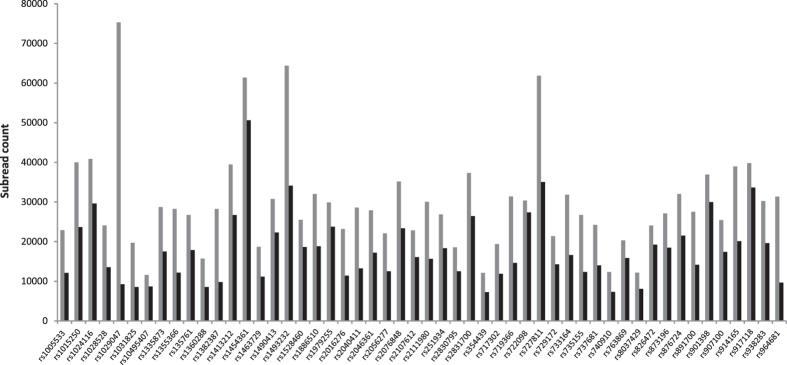
Number of extracted subreads per SNP locus (grey) and number of mapped subreads against the SNP reference sequence per SNP locus (black).

SNP amplicon subreads (see Data analysis for an explanation on subreads) were extracted from the high quality 2D reads, resulting in 1542739 sequences. Figure 2 displays the number of subreads per SNP locus (grey) as well as the number of subreads that uniquely mapped to the SNP reference sequences (black). The average number of subreads per SNP locus that could be extracted from the sequenced fragments was 29888 (SD = 12718), with a respective maximum and minimum of 75310 and 11592 extracted subreads. Considering the fact that the amplicons were pooled in equimolar quantity before random ligation and library preparation, the representation bias is considerable. With an average depth after mapping of the subreads of 17933 (SD = 8452), all loci have a depth that should be adequate for SNP calling. The average mapping rate of the subreads against their respective reference sequences was 60%. For some loci, the mapping rate was particularly low. Locus rs1029047 has the lowest mapping rate with only 12% of the rs1029047 subreads mapping to this locus. The discrepancy between the amount of extracted and the amount of mapped subreads is caused by the difference in methodology: Extraction of the subreads from the sequencing reads is done using only the primer sequences, whereas the mapping procedure attempts to align an inner subread region to the 51 nucleotide long SNP reference sequence and excludes subreads with a high number of mismatches. In the rs1029047 locus, a long homopolymer stretch of 10 consecutive adenine (A) bases results in nanopore sequences with a high number of errors in the length of the homopolymer stretch. This causes too many mismatches in the mapping step and thus yields a low number of mapped reads. Issues with sequencing homopolymeric stretches have been reported by several groups in the Nanopore community^8^: The MinKNOW software performs a non-linear filtering on the raw data, to provide a secondary data stream of events which all subsequent analyses (e.g. base calling) are based upon. The event extraction is complicated by the stochastic behavior of the DNA molecule, the nanopore complex and the translocation enzyme. This enzyme tends to randomly ratchet the DNA too slowly or quickly through the pore resulting in variable dwell times, thereby omitting event detection and resulting in a deletion in the final base called sequence^9^. This process is further complicated when analyzing long homopolymeric stretches, which show no distinct current changes (events) for an extended period while moving through the pore. Analogously, the rs1029047 locus has also proven difficult to sequence with Ion Torrent sequencing^6^,^10^,^11^, a sequencing technology that is also error-prone when sequencing homopolymers.

**Figure 3:**
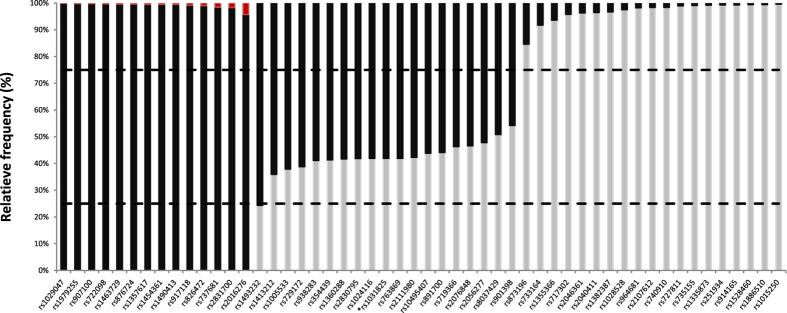
Relative frequency of mapped subreads containing one of the two possible SNP alleles (grey and black). Red bars show the proportion of reads containing an unexpected base at the SNP position. The allelic imbalance cut-offs are indicated by dashed lines. Loci discordant with the Illumina reference are indicated with an asterisk.

The relative frequency of mapped subreads per SNP locus containing either of the two possible alleles (black/grey), is shown in Fig. 3. The proportion of mapped subreads not uniquely mapping to either of the two possible alleles is shown in red. The allelic imbalance cut-offs (see Materials and Methods) are indicated by two horizontal dashed lines. Based on these cut-offs, 51 of the 52 SNPs loci had a genotype corresponding to the genotype produced by Illumina sequencing. These SNP profiles, generated by Oxford Nanopore and Illumina sequencing can be found in Supplementary Table 1. The incorrectly called rs1031825 locus resulted from allelic imbalance. The locus was classified as a heterozygous while the Illumina reference profile showed a homozygous genotype. Although the rs1493232 locus was called correctly as homozygous, it showed a severe allelic imbalance with 75.7% A and 24.3% C. Both the rs1031825 and rs1493232 loci have their analyzed SNP located between or inside homopolymer stretches that consist of the same bases as the reference and alternative base at the SNP position, respectively AA[A/C]CCCC and CC[C/A]CAAAA. Examination of the mapped sequences by IGV (a screenshot for each of the 2 loci is available as Supplementary Figure 1) indeed reveals that small sequencing errors, usually linked to short polymeric tracts of four or more bases, tend to produce false indels. Also when using other genotyping technologies, the rs1493232 and rs1031825 loci were reported to be problematic. Børsting *et al*. reported a substantial allelic imbalance of the rs1031825 locus using Ion Torrent sequencing^12^, a sequencing technology which is also error-prone when sequencing homopolymers. R. Daniel *et al*. reported discordant genotypes for the rs1493232 locus when comparing SNaPshot, Ion Torrent and Sanger sequencing^6^. Based on these observation other loci were screened for the potential of SNP misinterpretation due to adjacent homopolymer regions. Three additional loci (rs1029047, rs733164 and rs873196) were identified as vulnerable for misinterpretation (a screenshot for each of the 3 loci is available as Supplementary Figure 2). Two of which (rs1029047 and rs733164) had already been described as challenging to sequence correctly because of homopolymer regions by Børsting *et al*.^12^.

The use of a special BWA setting (ONT2D), specifically designed for ONT datasets and allowing to handle a number of typical Oxford Nanopore sequencing errors, was proposed to produce better alignments. Although this special setting mapped more subreads and raised the average coverage depth per locus, the same loci remained problematic. A negative effect of this relaxed alignment setting was that the overall alignment quality dropped substantially, with more mismatches and indels being observed. This reduced alignment quality further hampered the correct SNP calling. Supplementary Figures 3 and 4 are based on the analysis using BWA mapping with the ONT2D setting, showing the results corresponding to Figs 2 and 3. A more likely improvement would be to exclude or substitute the most problematic homopolymer containing loci in the SNP panel. This would result in a more robust genotyping.

The newest version of the MinION flow cell (R9.4) yielded a number of reads that is much higher than needed for SNP calling in one sample. This opens the possibility to multiplex several samples in one run. To explore this, we downsampled the fastq files to 1/100^th^ and redid the analysis: The same SNP profile was generated (available as Supplementary Figure 5) as with the full dataset.

The data was deposited in the European Nucleotide Archive (ENA) database under project accession number PRJEB18110 (http://www.ebi.ac.uk/ena/data/view/PRJEB18110).

## Materials and Methods

### Pcr Amplification

The results presented in this paper were obtained using sample 9947 A, a female single contributor control DNA sample (Promega, Madison, US). The 52 SNP containing regions were individually amplified via PCR using a protocol based on the forensic SNP multiplex developed by the SNPforID consortium^1^. The primers were used according to the designer’s specifications (primer sequences available in Supplementary Table 2). PCR was performed in 25 μl containing 2.5 ng template DNA, 1 × PCR buffer (Qiagen), 3 mM MgCl_2_, 8 μM dNTPs (Thermo Fisher Scientific, Waltham, USA), 2 U HotStar Taq polymerase (Qiagen) and 0.5 μM of both forward and reverse primer. The temperature profile consisted of an initial denaturation at 95 °C for 15 min, followed by 35 cycles including denaturation at 95 °C for 30 s, primer annealing at 60 °C for 30 s, extension at 72 °C for 30 s and a final elongation step of 10 min at 72 °C. The *Agilent High-Sensitivity DNA kit* (Bioanalyser, Agilent Technologies, California, USA) was used to assess the quality of the generated PCR products.

### Nanopore Sequencing

ONT’s Metrichor base calling software currently requires DNA fragments to have a minimum length of 100 bp to be processed. To comply with this restriction, the individual PCR amplicons were pooled and randomly concatenated via ligation to create longer DNA fragments. To accomplish this, we first purified the individual PCR products via gel electrophoresis in order to remove excess primers and enzyme (E-gel 2%, Thermo Fisher). The fragments of interest were recovered by cutting out the 59–115 base pairs (bp) region of the gel and processed using the Zymoclean Gel DNA Recovery Kit (Zymo Research, Irvine, USA). The purified amplicons were quantified fluorimetrically using a Qubit fluorimeter (Life Technologies, Paisley, UK) and pooled in equimolar quantities. The fragments in the mixture were end-polished using the NEBNext End-Repair module (NEB, Ipswich, USA). Random concatenation of the amplicons was performed using the Blunt T/A Ligase Mastermix (M0367S NEB, Ipswich, USA). The ligation reaction proceeded for 45 min and the DNA was subsequently recovered using 1.8 volumes of AMPure XP beads (Beckman Coulter, High Wycombe, UK). The quality of the ligation products was assessed using the *Agilent High-Sensitivity DNA kit* (Bioanalyser, Agilent Technologies, California, USA).

Oxford Nanopore sequencing requires the attachment of the ONT specific leader and hairpin (HP) adaptor to the sample. End-repair was performed on 1.034 μg of the concatenated amplicons using the Ultra II End-Repair/dA-Tailing module (NEB, Ipswich, USA) according to the manufacturer’s instructions. The resulting A-tailed DNA was cleaned-up using 1.8 volumes of AMPure XP beads (Beckman Coulter, High Wycombe, UK) according to the manufacturer’s instructions. Then, 8 μl nuclease free water, 10 μl Adaptor Mix and 2 μl HP adaptor were added to the eluate, followed by 50 μl of Blunt/TA ligase master mix (NEB). Between each sequential addition the library was mixed by inversion. The reaction was incubated at room temperature for 10 min after which 1 μl of HP tether was added. The reaction was left to proceed for another 10 min at room temperature. This adaptor-ligated, tether-bound library was purified using 500 ng of MyOne C1 Dynabeads (Thermo Fisher, UK) and recovered in 25 μl elution buffer. Finally, the library was quantified fluorimetrically using a Qubit fluorimeter (Life Technologies, Paisley, UK). This so called pre-sequencing mix had a final DNA concentration of 6.84 ng/μl. The sequencing mix was prepared by adding 37.5 μl of running buffer and 37.5 μl of library loading buffer to 12 μl of pre-sequencing mix. This library was loaded in dropwise fashion via the Spot-ON port on an R9.4 Spot-ON flow cell. Sequencing (protocol 48-hour FLO-MIN106_SQK-LSK208) was stopped after a 24 h non-stop run. The flow cell was topped up twice (after 6 h and 12 h) with freshly made sequencing mix.

### Data Analysis

The raw data generated by the MinION device was processed by ONT’s cloud based Metrichor service, which performs base calling using a Recurrent Neural Network (RNN). The sequencing data of the concatenated amplicons were retrieved as a set of *fast5* files. The individual amplicon sequences were excised from the reads using a custom python script consisting of the following steps: (1) Sequences and their quality scores were extracted from the *fast5* files and written into a *fastq* file. (2) Using Python’s regular expression module (regex, a functionality to construct search patterns) and the PCR primer sequences (Supplementary Table 2), individual SNP amplicon sequences within each sequencing read were identified and collected as subreads. To take into account nanopore sequencing errors, the regular expressions allowed up to 3 mismatches in each primer region. (3) Finally, subreads with a length between 50 and 150 nucleotides were collected. Detailed documentation including the python script is available on the SNPore Github repository (https://github.com/SenneC1/SNPore). All subreads were aligned against the reference sequences of the 52 SNP loci (consisting of the SNP and 25 nucleotides of flanking region on either side) using the BWA (version 0.7.15) software (Burrow-Wheelers Aligner)^13^ and default settings. The alignment data was used to generate a table of all nucleotide variations at all positions by the SAMtools (version 1.3.1)^14^ and BCFtools (version 1.3.1)^15^ software, allowing detection and quantification of the SNP alleles. Documentation and scripts can be found at the *SNP detection* notebook on the SNPore GitHub repository (https://github.com/SenneC1/SNPore). When > 75% of the reads corresponded to the reference allele with the remaining reads corresponding mainly to the alternative allele, a homozygous call was made. This arbitrary rule will be further referred to as the allelic imbalance cut-off. Finally a visual inspection of the subread alignments was executed using IGV viewer^16^ in an attempt to trace erroneous mapping.

### Reference Profile

A reference profile of the SNPforID loci for the 9947 A sample was created by Illumina sequencing. The 9947 A reference sample was amplified in a single multiplex according to Sanchez *et al*.^1^. A library of this amplified sample was created using *NEBNext^®^ Ultra DNA Library Prep* (New England Biolabs, Ipswich, USA), according to the manufacturer’s protocol: The Illumina TruSeq DNA Sample Preparation Kit (Illumina, San Diego, USA) was used to add adapter sequences to the ends of the DNA fragments, followed by a MinElute PCR Purification (Qiagen) procedure to remove excess buffer and enzyme. Size selection was performed with the E-Gel iBase Power system (Invitrogen) using an E-gel EX 2% agarose gel and a 1 kb Plus DNA ladder (Thermo Fisher). Fragments with a size of approximately 180–300 bp (amplicon + adapters) were cut from the gel and purified using the *Zymoclean gel DNA recovery kit* (Zymo research). The recovered DNA fragments were then subjected to an Agilent Bioanalyzer chip analysis (Agilent Technologies) to ensure that the adaptor ligation was successful. The exact amount of sequence-able library fragments was determined by performing a qPCR using the *Sequencing Library qPCR Quantification* kit (Illumina, San Diego, USA). Finally, single-end index 75 bp sequencing was performed on a high output flow cell on a NextSeq 500 (Illumina). The resulting sequencing reads were aligned against the reference sequences of the 52 SNP loci (consisting of the SNP and 25 nucleotides of flanking region on either side) using the BWA software (version 0.7.15). Variant calling and determination of the SNP alleles was done as described for the nanopore sequencing.

## Conclusion

This study demonstrates proof-of-concept forensic SNP genotyping using the Oxford Nanopore MinION sequencing platform and shows the current capabilities of the system. Current minimum amplicon length limitations of the technology can be circumvented by applying random amplicon ligation before the library preparation, combined with bioinformatical retrieval of the amplicon sequences as subreads. All but one of the 52 loci were genotyped correctly. We identified two SNP loci that prove to be problematic to genotype robustly using nanopore sequencing. Both problematic loci contained homopolymers in the sequence flanking the forensic SNP and were already reported as problematic in studies using other sequencing technologies. When these loci are avoided, correct forensic genotyping using nanopore sequencing is technically feasible. The total sequencing throughput of the MinION R9.4 flow cell should allow to multiplex dozens of forensic samples on one flow cell. The technique is however still subpar compared with current techniques such as capillary electrophoresis and Illumina sequencing in terms of costs, analysis time, sequence error rate, representation bias and allelic imbalance. With the ongoing improvements, nanopore sequencing may become suitable for routine use in the future.

## Additional Information

**How to cite this article**: Cornelis, S. *et al*. Forensic SNP Genotyping using Nanopore MinION Sequencing. *Sci. Rep.* **7**, 41759; doi: 10.1038/srep41759 (2017).

**Publisher's note:** Springer Nature remains neutral with regard to jurisdictional claims in published maps and institutional affiliations.

## Supplementary Material

Supplementary Information
