# Supplementary material for: Forensic SNP Genotyping using Nanopore MinION Sequencing
Source: Sci Rep. 2017 Feb 3;7:41759. doi: 10.1038/srep41759 (PMC5290523; doi:10.1038/srep41759)
Supplement: Supplementary Information [file srep41759-s1.pdf]

Forensic SNP Genotyping using Nanopore MinION Sequencing

AUTHORS

Senne Cornelis[1], Yannick Gansemans[1], Lieselot Deleye[1], Dieter Deforce[1,#],Filip Van Nieuwerburgh[1,#,*]

[1] Laboratory of Pharmaceutical Biotechnology, Ghent University

# Contributed equally

* Corresponding author at: Laboratory of Pharmaceutical Biotechnology, Faculty of Pharmaceutical Sciences, Ghent University, Ottergemsesteenweg 460, Gent, Belgium.

Supplementary Information

	Oxford Nanopore		Illumina	
rs2056277	C	T	C	T
rs1413212	A	G	A	G
rs2107612	G	G	G	G
rs2111980	A	G	A	G
rs251934	T	T	T	T
rs1028528	G	G	G	G
rs2831700	A	A	A	A
rs901398	C	T	C	T
rs722098	A	A	A	A
rs2076848	A	T	A	T
rs1493232	A	A	A	A
rs735155	G	G	G	G
rs1528460	T	T	T	T
rs1005533	A	G	A	G
rs733164	G	G	G	G
rs1029047	A	A	A	A
rs727811	C	C	C	C
rs1024116	A	G	A	G
rs1015250	G	G	G	G
rs907100	C	C	C	C
rs737681	C	C	C	C
rs717302	G	G	G	G
rs740910	G	G	G	G
rs1979255	C	C	C	C
rs719366	C	T	C	T
rs2040411	G	G	G	G
rs2016276	A	A	A	A
rs135761	A	A	A	A
rs8037429	C	T	C	T
rs1335873	T	T	T	T
rs876724	C	C	C	C
rs354439	A	T	A	T
rs1031825 *	A	C	C	C
rs873196	T	T	T	T
rs1463729	A	A	A	A
rs1886510	T	T	T	T
rs763869	C	T	C	T
rs2830795	A	G	A	G
rs1454361	A	A	A	A
rs1355366	G	G	G	G
rs938283	C	T	C	T
rs1490413	A	A	A	A
rs964681	T	T	T	T
rs826472	C	C	C	C
rs729172	A	C	A	C
rs1382387	T	T	T	T
rs10495407	A	G	A	G
rs891700	A	G	A	G
rs917118	C	C	C	C
rs914165	G	G	G	G
rs2046361	T	T	T	T
rs1360288	C	T	C	T

Supplementary Table 1: Sample 9947A SNP profiles generated with Oxford Nanopore and Illumina sequencing. Discordant loci are indicated by a red asterisk.

	Forward primer sequence	Reverse primer sequence
rs1490413	GTGTGGACTGGGCTGATGT	TTCTCACTAGTGTCCCTGCTCTG
rs876724	GCAGGCTCCATTTTTATACCACT	GAATATCTATGAGCAGGCAGTTAGC
rs1357617	AGCTGATGCAGACCACTTCAC	GGATAGCTGATAAGAAACATGACCA
rs2046361	CCTATTTGTATGTATCTATTGTCTATGAACG	GTCATTGTTGACACTTCACCTTCTA
rs717302	CTTTAGAAAGGCATATCGTATTAACTGTG	AACACAGAAAGAGGTTCATATGTTGG
rs1029047	CATAACGTGGATTTGTCAGCA	GGAATAAACTGAAGGCTAAAGAAAAG
rs917118	GCCCTTTAGGGTCGGTTC	GTAAGAGATGACTGAGGTCAACGAG
rs763869	ATCAAGTGCTTTCTGTTGACATTTG	GGCTACTCCCTCATAATGTAATGC
rs1015250	AAGTGATGGAGTTAGGAAAAGAACC	AAGACATTAGGTGGATTCATAGCTG
rs735155	GGAGAAAACCGGAGAGCTG	GAGTGTCACCGAATTCAACG
rs901398	CTGGGTGCAAACTAGCTGAATATC	CTGGAATGTACTAGGCAAGAAACTAA
rs2107612	GAGCATTCTCTTCTGTTAAAATTGC	TGAGTACATTATTCAACTGTTTTGGAG
rs1886510	GTCCTTGTCAATCTTTCTACCAGAG	GGATTTTCACAACAACACTTGC
rs1454361	AGGGAAATACACCCTGAGCTG	AGCTGTCCATCATCAGTAAGACAC
rs2016276	TGCATCCCAGCCTCCACT	ATTGTACCTTGCCACTTTGTGTG
rs729172	CATTAATATGACCAAGGCTCCTCT	ACATTTCCCTCTTGCGGTTAC
rs740910	GTATAACAGTTTGCTAAGTAAGGTGAGTG	AGATAGGTTCGAGTTTTGGCTTTA
rs1493232	CTATTCTCTCTTTTGGGTGCTAGG	CAAACTGTTTATTGTGAGGCCTGT
rs719366	CACAGCATCTTTTAACTCTTTTATTATCC	GTAAGGACTTATAGTGAGTAAAGGACAGG
rs1031825	CTTATCTTTCCCACATTATGGTCCT	AAGATATAATCACTGCTTTCAAGTATGC
rs722098	GGAAGTACACATCTGTTGACAGTAATGA	GGGTAAAGAAATATTCAGCACATCC
rs733164	AGCTTTCAGCCCCAGGTC	CGGCTCAGGAATGTCAGG
rs826472	TGAATTTTGTCTCTGTTATATTAGTCACC	TGTAATTGAAATTTGTAGGCAATAGAC
rs2831700	GGCTAAACTATTGCCGGAGA	TTCCCTAGAACCACAATTATCTGTC
rs873196	GCATTCAAATCCCAAGTGCT	GCAGGAGTTGGAGTCAATCAG
rs1382387	ACGAAGGAGAAACACCTGAACT	TGGAGTACTTAATAAGACGCTGCAT
rs2111980	AGCATCTTGGCAGCATCC	AGCAAGATCTTTGCCAGTGAGT
rs2056277	CCAAACTGGGTGTTAGGGAGAC	TCATTATCTCGTCATACTTCCCTGT
rs1024116	CCATGTGTTCTAATAAAAAGGATTGC	TGGGAAGTGAGCAAAAGTAAATACA
rs727811	GTGTTTCTTTTTCTCTTACCGGAAC	GTGAATGAAATCATGAGATTGCTG
rs1413212	AACCTCCTTTGGAAACACTGAC	CAACATTCCATTATCCAGGAGAC
rs938283	CATTGAAGTCCTAACCCCTAGTACG	GGATGAGGCCCACCCATA
rs1979255	TCAGAGACTATGGATGGTATTTAGGTC	CATGGAACGTTGGAACTCTTG
rs1463729	ACTATCAGTCTCTGCCCTTATTCTG	CACATGTGCATGCTTTTGG
rs2076848	GCCTCACCACCAGAAATCAG	GACATCAGAAACTCCCATGAAACT
rs1355366	CCATGATTTTCTTGTGGTGAGA	CACATGTGCTTAGGCCACAAC
rs907100	GGAGTTCCTGATAACGATTCTGAAG	ACAGAAAAGAAGCCCAGTTGGA
rs354439	GGCTTCTCTTTCCCTTATGTATCTC	CAGGTTGCGATAGAAAACAGTGAAT
rs2040411	TCTGGAATGCCAGTTCTTTTGT	CAGAACGCCTATGAAAACCAGT
rs737681	ACATGTGAGGCCATCTCCAC	CCTTACTGTGATGTAGGCACTGTTC
rs2830795	CACTTCTATAGACATAGGACACACCAT	ATCTAGGCTCTGAATCAGGATGAG
rs251934	AGAGGGCAGTGAGGCTTTTAAGTAG	TGCTAGAATCCAGACTTAACTACCAG
rs914165	AGCAGCAGAGCCTGGATG	AGACCAGTCACCTCTTTTGCACT
rs1049540	AGATCTCCACTTCCTCTTGGTTG	CTCCCAAATTTACATTGCCACT
rs1360288	AGACTCTCTGTGTGTGGCTTTG	GAGGGGGCATCTGTTGAG
rs964681	GTACCTGGAGGTGATTTCTGTGAG	GTTATGGAGGATTGGTAAGAACCAG
rs1005533	GGTTTGTGTGTGAGTGTTTCAGAT	CCTTATGCCTCCCCTGAAC
rs8037429	TTCACTTTGCTACACCTCCATAGTA	TGCTACGTAAGAGGTCATTGCTATC
rs891700	TTTTCAGAGGTGGTATTTCTAGCTG	GCTATGACACTCCTTAGAACTATGCAA
rs1335873	GTGGATGATATGGTTTCTCAAGG	TTCAACAAACGTGTGATGCTCT
rs1028528	ACAGCTGATGCCTCCCTGA	GAGGATGAAGGTTAGAGCCAGAC
rs1528460	TCCTGGAGATCAATATTTAGCCTTA	GGGTGACCAGTAGTTCTATGAGC

Supplementary Table 2: Forward and Reverse PCR primer sequences for the 52 SNPforID loci

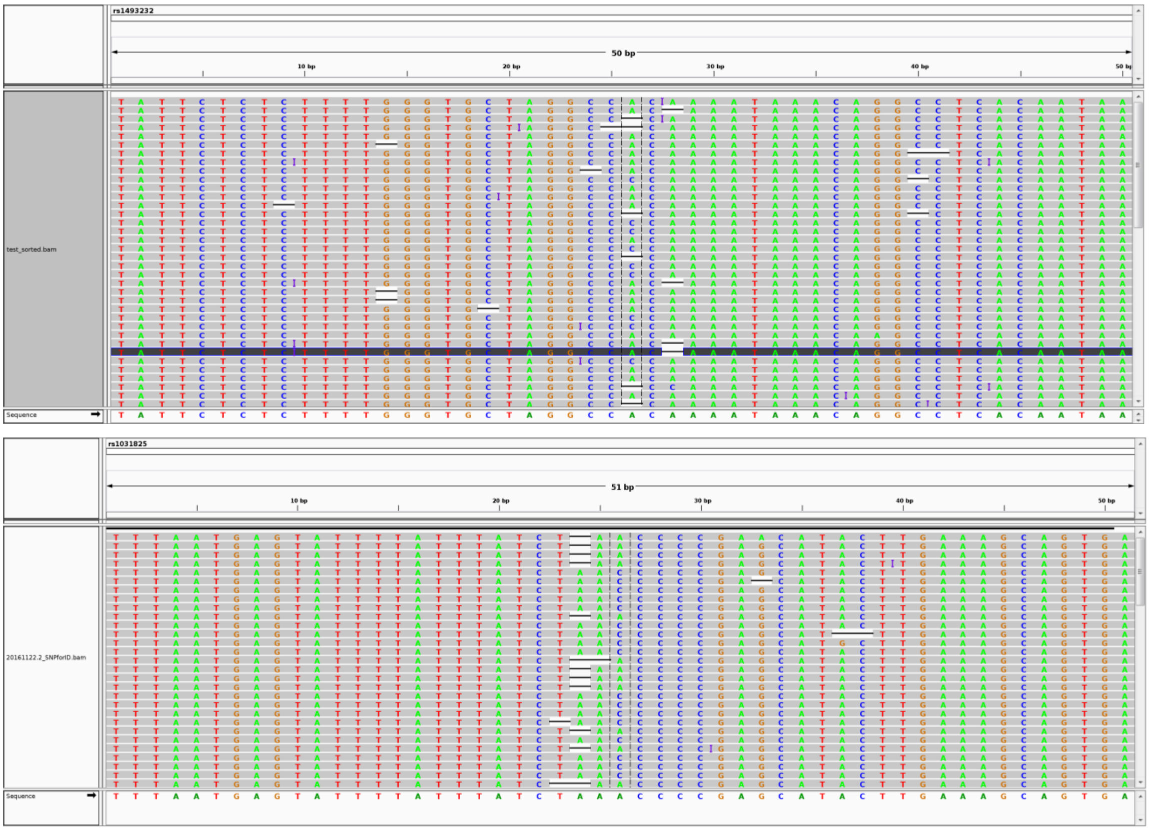

Supplementary Figure 1: IGV screenshots of the mapped reads at locus rs1493232 (top) and rs1031825 (bottom)

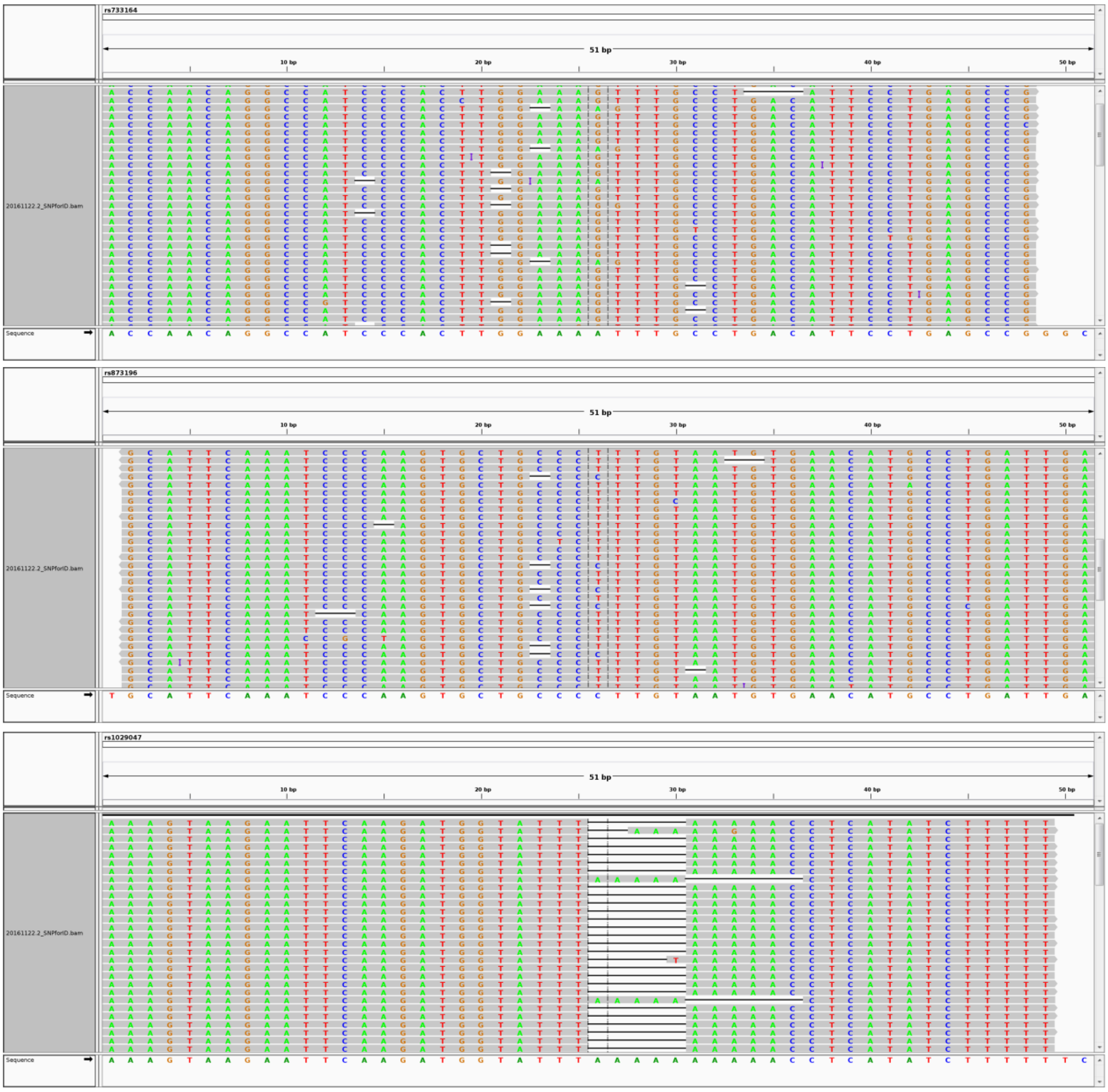

Supplementary Figure 2: IGV screenshots of the mapped reads at locus rs733164 (top), rs873196 (middle) and rs1029047 (bottom).

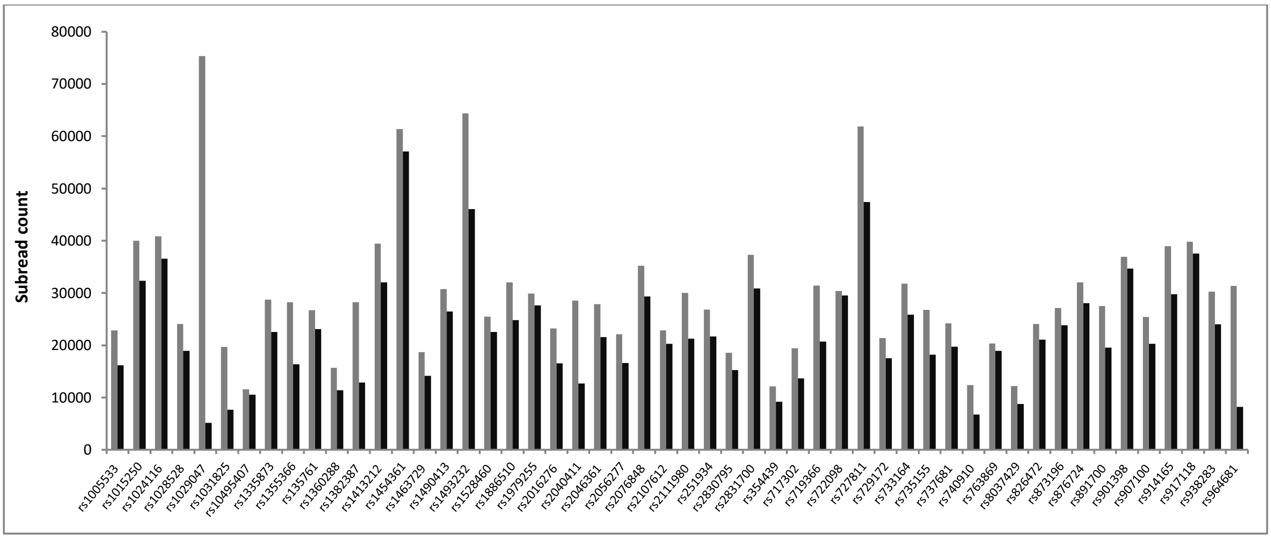

Supplementary Figure 3: Number of extracted subreads per SNP locus (grey) and number of mapped subreads against the SNP reference sequence per SNP locus (black) using the BWA ONT2D setting.

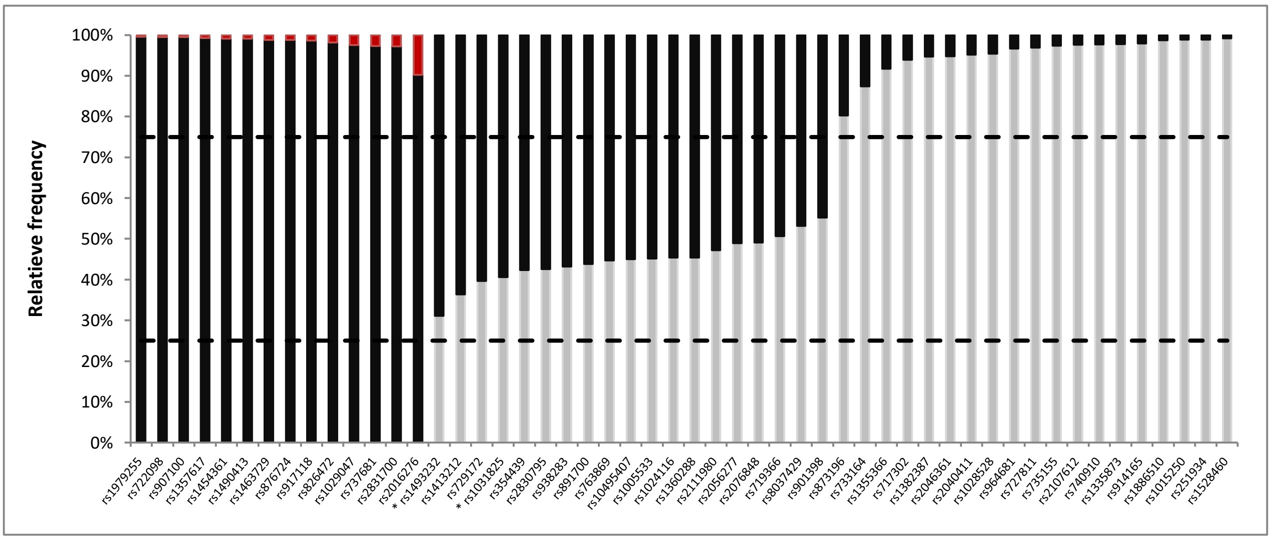

Supplementary Figure 4: Relative frequency of mapped subreads using the BWA ONT2D setting containing one of the two possible SNP alleles (grey and black). Red bars show the proportion of reads containing an unexpected base at the SNP position. The allelic imbalance cut-offs are indicated by dashed lines. Loci discordant with the Illumina reference are indicated with an asterisk.

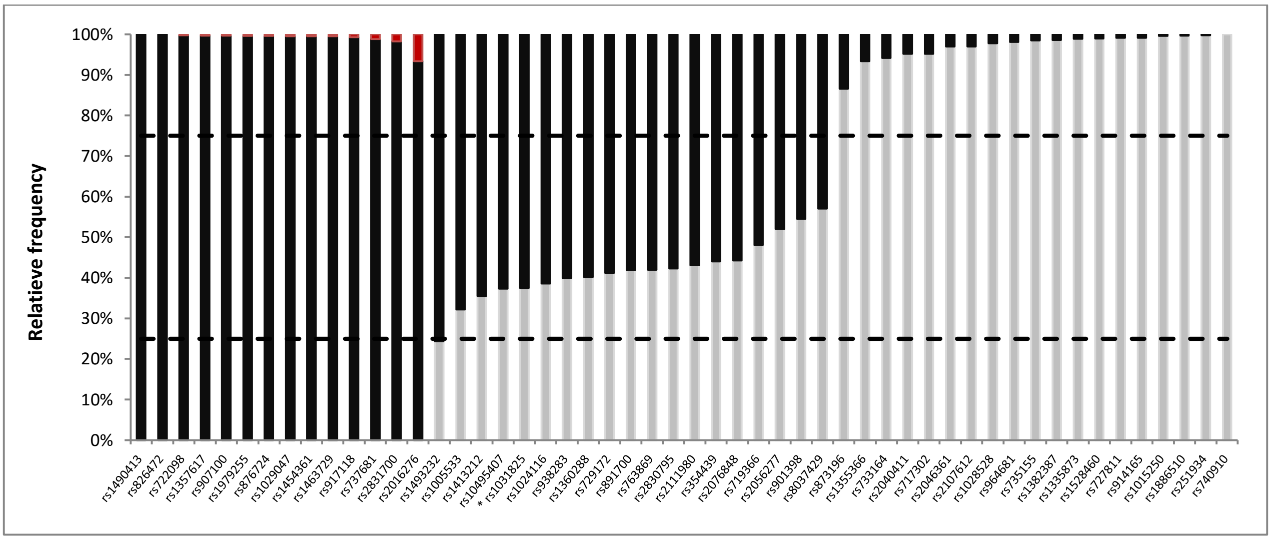

<u>Supplementary Figure 5:</u> Relative frequency of mapped subreads using 1 percent of the available data containing one of the two possible SNP alleles (grey and black). Red bars show the proportion of reads containing an unexpected base at the SNP position. The allelic imbalance cut-offs are indicated by dashed lines. Loci discordant with the Illumina reference are indicated with an asterisk.
